# Aortoenteric Fistula as a Complication of Open Reconstruction and Endovascular Repair of Abdominal Aorta

**DOI:** 10.1155/2014/383159

**Published:** 2014-09-14

**Authors:** Marek Tagowski, Hendryk Vieweg, Christian Wissgott, Reimer Andresen

**Affiliations:** Institute of Diagnostic and Interventional Radiology and Neuroradiology, Westkuestenklinikum Heide, Academic Teaching Hospital of the Universities of Kiel, Luebeck and Hamburg, Esmarchstraße 50, 25746 Heide, Germany

## Abstract

The paper intends to present a review of imaging characteristics of secondary aortoenteric fistula (AEF). Mechanical injury, infection, and adherence of a bowel segment to the aorta or aortic graft are major etiologic factors of AEF after open aortic repair. The pathogenesis of AEF formation after endovascular abdominal aortic repair is related to mechanical failure of the stent-graft, to stent graft infection, and to persistent pressurization of the aneurysmal sac. The major clinical manifestations of AEF comprise haematemesis, melaena, abdominal pain, sepsis, and fever. CT is the initial diagnostic modality of choice in a stable patient. However, the majority of reported CT appearances are not specific. In case of equivocal CT scans and clinical suspicion of AEF, scintigraphy, ^67^Ga citrate scans or ^18^F-FDG PET/CT is useful. Diagnostic accuracy of endoscopy in evaluation of AEF is low; nevertheless it allows to evaluate other than AEF etiologies of gastrointestinal bleeding. Without adequate therapy, AEF is lethal. Conventional surgical treatment is associated with high morbidity and mortality. The endovascular repair may be an option in hemodynamically unstable and high-risk surgical patients. We also illustrate an example of a secondary AEF with highly specific albeit rare radiologic picture from our institution.

## 1. Introduction

Secondary aortoenteric fistula (AEF) is a rare but potentially lethal complication of aortic surgery first reported by Brock in 1953. This currently better-known pathology still poses considerable diagnostic difficulties due to its unspecific clinical and radiologic manifestations. The aim of this paper is to present the current diagnostic approach to secondary AEF taking into account its complex and heterogeneous pathogenesis as a cause of the broad spectrum of its radiologic manifestations.

For a better understanding of the subject, we commence the paper by presenting a secondary AEF with highly specific albeit rare radiologic picture from our institution.

A 73-year-old man was referred to our emergency department with severe hematemesis and melena, suggestive of gastrointestinal bleeding. Low blood pressure and tachycardia indicated circulatory shock; laboratory results revealed anemia and elevated inflammatory markers (leukocytes 16.8/nL, CRP 14.5 mg/dL). In the medical history it was noted that he survived a covered perforation of an abdominal aortic aneurysm 2 months earlier. The incident was treated in another hospital with an open surgical approach, using a rifampicin-soaked Dacron Y-prosthesis. No complications occurred during the hospitalization but the patient refused to undergo follow-up examinations.

**Figure 1:**
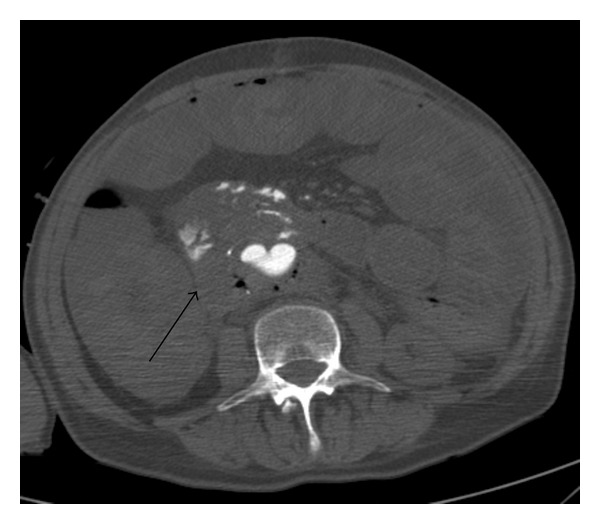
Axial contrast-enhanced CT scan of AEF.

**Figure 2:**
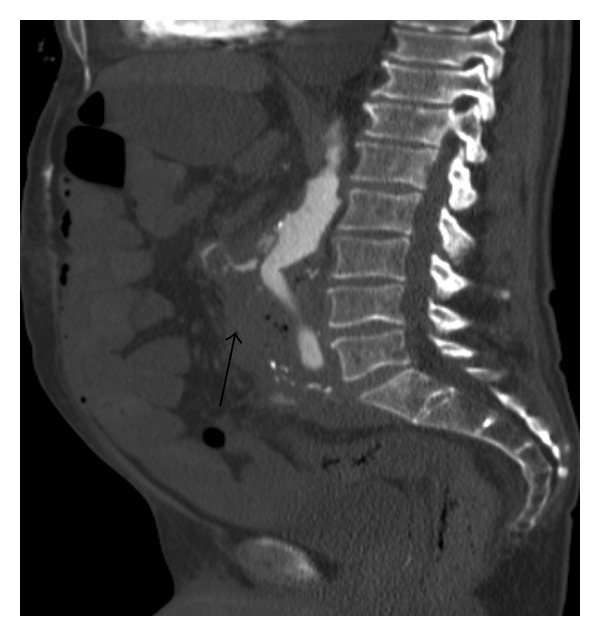
Sagittal contrast-enhanced CT scan of AEF.

**Figure 3:**
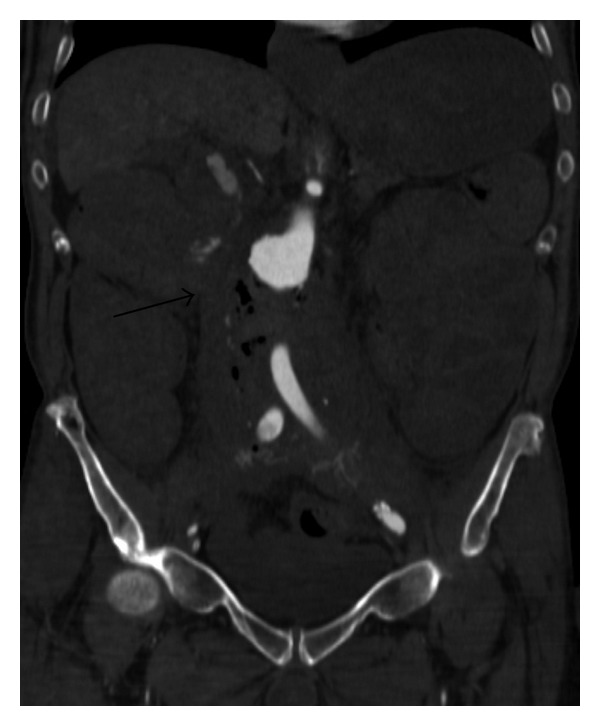
Coronal contrast-enhanced CT scan of AEF.

We performed a CT angiography of the abdominal aorta without oral or rectal contrast. The scans revealed a narrow fistulous communication between aorta and the third part of the duodenum with active extravasation of contrast-enhanced blood from the aortic to the duodenal lumen (Figures 1, 2, and 3).

Only a few minutes later the patient passed away due to uncontrollable blood loss.

In Figures 1–3, CT scans show extravasation of contrast material from the aorta into the third part and second part of the duodenum, perigraft gas collections, and increased soft tissue and fluid between the stent-graft and aortic wall and in the periaortic location.

## 2. Review

Secondary aortoenteric fistula may be a complication of open surgical repair as well as endovascular interventions of the aorta. It consists in development of communication between aortic lumen and the gastrointestinal tract.

The age distribution of the reported patients with diagnosed secondary AEF is broad with the median around 65 years [1]. The majority of afflicted patients are male [1].

Its annual incidence approximates 1% [1] after elective procedures and 14% [2] after emergency repair of ruptured aneurysm, which corresponds to increased likelihood of microbiological contamination of the periaortic tissues and bowel trauma during hurried surgical dissection in the latter setting [1, 2].

The predominant original aortic procedures that can give rise to this complication are the elective abdominal aortic aneurysm resection, aortic replacement or bypass for aortoiliac occlusive disease, resection of ruptured aneurysm, and stent graft placement for aortic aneurysm [1, 2].

The most predisposed to fistula formation gut segments are the third and fourth portions of duodenum [2, 3] because of their retroperitoneal location and anatomic relationships with the graft. Less frequently other intestinal segments including stomach and appendix could also be involved in AEF formation [1, 2].

There were also reported rare cases with more than one simultaneous fistulization.

The AEF most frequently occurs at the proximal suture line (in the substantial number of patients with frank pseudoaneurysm formation), and graft body and distal anastomosis are less common locations [1, 2].

Median interval from the original operation to the formation of AEF is between 24 and 47 months with a broad time range from 2 days to 26 years [1].

Mechanical injury and infection may each act as a major etiologic factor of AEF.

Bowel trauma by surgical dissection or due to direct pressure of anastomotic pseudoaneurysm as well as direct pulsatile pressure by a graft predispose to focal necrosis and erosion of bowel wall. This enables local infection of adjacent graft material with resultant disruption of the vascular anastomosis and exsanguinating hemorrhage [3].

The infection may be caused by contamination from the environment at the time of the original operation, which is suggested by the presence of cutaneous flora (*S. aureus* or* S. epidermidis*) in the intraoperative cultures [3, 4].

By contrast, occurrence of Enterobacteriaceae and anaerobic bacteria is compatible with the secondary contamination of the graft material from the intestinal flora.

The third postulated source of infection may be postoperative bacteriemia from any cause (including transient bacteriemia from dental procedures or gastrointestinal endoscopy).

Graft susceptibility to bacteriemia is highest during the first postoperative months and decreases with formation of neointima and healing of the anastomotic sutures [2, 4].

Regardless of these factors adherence of a bowel segment to the aorta or aortic graft is essential for development of the aortoenteric communication [3, 4].

Therefore adequate tissue coverage and reperitonealization of the graft during the original operation are of utmost importance.

Relatively rare diagnosed fistulous communication between the bowel lumen and periaortic tissues is termed paraprosthetic-enteric fistula and is regarded as a step in the development of aortoenteric fistulization [3, 4].

The pathogenetic mechanism of AEF development after endovascular abdominal aortic repair may be related to mechanical failure of the stent-graft such as rupture or migration with kinking of the device in the aneurysmal sack, which leads to aortic graft erosion and pressure necrosis of the bowel wall [5–7].

AEF formation can also result from stent graft infection that predisposes to erosion of the adherent bowel wall [8, 9] as well as from persistent pressurization of the aneurysmal sack in the presence of endoleak or endotension [5, 10–12] (including endotension secondary to infection in an excluded aneurysm sac [10, 13]).

As holds true for the graft infection after surgical aortic repair, stent-graft infection may also be a result of AEF [14–16].

A rare cause of AEF development after endoleak embolization may be chronic injury of the aortic wall from metallic coils embedded in the aortic wall [17, 18].

In addition, aortoduodenal fistulization is precipitated by periaortic inflammatory tissue after endovascular repair of an inflammatory aortic aneurysm [15].

The major clinical manifestations of AEF are hematemesis (41%) and melena (54%) (with hemodynamic shock during the course of medical management in almost one third of cases) as well as abdominal pain, sepsis, and fever which occur, respectively, in 21%, 12%, and 11% of the afflicted patients [1].

Other less frequent presenting symptoms include back pain, pulsating tumor in abdomen, and anorexia [1].

Gastrointestinal herald bleeding which precedes the exsanguinating hemorrhage was reported in approximately 54% of cases [1].

It has been explained either as a result of a small fistulization temporarily sealed by thrombus or initial bowel wall ulceration.

Of particular importance is the fact that not every patient with AEF presents with detectable gastrointestinal bleeding.

Taking into consideration its devastating consequences there is no single, satisfyingly reliable diagnostic method of AEF. Even diagnostic laparotomy in a retrospective study has strikingly yielded an accuracy of “only” 91% [1], which underscores the importance of a strong clinical suspicion of possible aortoenteric fistulization in a patient after aortic procedures.

Nowadays computed tomography is regarded as the initial diagnostic modality of choice in a sufficiently stable patient, because of its high sensitivity and specificity (resp., 94% and 85% according to Low et al. [19]) in detecting perigraft infection with or without AEF and its wide availability in the emergency setting [19, 20].

The possible CT presentations of AEF are highly diverse. Therefore, the familiarity with the varied potential CT abnormalities reflecting this pathology is prerequisite for the prompt and accurate diagnosis.

Depicting of extravasation of contrast material from the aorta into the adjacent bowel lumen, leakage of gastrointestinal contrast agent into the periaortic space, and visualization of aortic graft in bowel lumen represent highly specific but very rare CT signs of AEF [21, 22].

The majority of its reported CT appearances are not specific. They are similar to those observed in perigraft infection (PGI) and include perigraft gas collections or gas in the graft lumen (>4–7 weeks following aortic reconstruction), focal thickening of the bowel wall adjacent to the aortic graft, periaortic soft tissue or fluid (>3 months following aortic reconstruction), focal disruption of the calcified aortic wrap, pseudoaneurysm formation, increased soft tissue between the graft and aortic wrap, and obliteration of the fat plane between the aorta and intestine. Additionally, rare reported CT findings of AEF are intramural duodenal hematoma and dystrophic vascular graft calcification in the bowel lumen [19–21, 23, 24].

According to Low et al. [19] the presence of ectopic gas collections and focal bowel wall thickening is more likely related to AEF than PGI without AEF.

In the postoperative period, periaortic hematoma should resolve within 3 months.

Ectopic gas collections are in most cases not detectable beyond the first postoperative week and should completely resolve within 4–7 weeks. After these periods any perigraft soft tissue, gas, or fluid should be presumed to be the sign of perigraft infection [25, 26].

In the first month following endovascular repair of abdominal aortic aneurysm perigraft gas within the aneurysmal sac may be a normal finding due to introduction of atmospheric air bubbles with the delivery system of the stent-graft [11, 27, 28].

Concurrent occurrence of these CT findings with fever and leukocytosis in the first days after stent-graft placement may result from local inflammatory response to the implanted stent-graft material without underlying infection, referred to as postimplantation syndrome.

During graft incorporation process after open aortic reconstruction hematoma between the graft and aneurysm wrap should gradually resolve. The study of Qvarfordt et al. presents its almost complete resolution in 7 weeks, with the aneurysm wall in close apposition to the graft [25]. Demonstration of increased soft tissue (>5 mm) between the graft and aortic wall can be the only CT finding suggesting graft infection.

The study of Hughes et al. [20] emphasizes the high diagnostic specificity related to the combination of gastrointestinal blood loss and the reported CT signs of AEF (periaortic fluid/soft tissue, breach of the aortic wall, pseudoaneurysm formation, loss of fat pad between aorta and intestine, ectopic gas, and intravasation of contrast material into the intestinal lumen).

Despite the limitation of this study due to absence of PGI cases without AEF in the control group, diagnostic significance of concomitant occurrence of gastrointestinal bleeding and CT characteristics of perigraft infection is unquestionable.

A significant number of surgically proven PGI (with or without AEF) demonstrates only subtle or no CT abnormalities [19]. This observation underscores the importance of meticulous evaluation of CT scans. In case of any doubt additional examinations including scintigraphy, MRI, or CT-guided percutaneous fine-needle aspiration biopsy may reveal the diagnosis.

Use of oral contrast material is frequently necessary to reliably assess the focal thickening of the bowel wall and allows to detect its extravasation into the periaortic space as a sign of AEF or paraprosthetic-enteric fistula [19, 20]. However, bowel opacification with positive contrast material is likely to obscure the intravasation of aortic contrast into the intestine.

Other conditions with overlapping CT features including retroperitoneal fibrosis and adenopathy should be also included in the differential diagnosis of secondary AEF.

Scintigraphy with autologous leukocytes labeled with ^99m^Tc-HMPAO or ^111^In and ^67^Ga citrate scans may be useful in case of equivocal CT scans and clinical suspicion of perigraft infection, when subtle morphological changes (e.g., after surgery) are indistinguishable from low-grade inflammation on CT scans [29–31]. Scintigraphy with ^99m^Tc-labeled autologous erythrocytes or ^99m^Tc-HMPAO-labeled leukocytes enables to depict extravasation of tagged cells into the bowel loops, a finding highly suggestive of AEF [32, 33].

Compared to scintigraphy, ^18^F-FDG PET/CT provides significantly more accurate anatomic localisation of infectious processes. It enables image acquisition within 1 hour after radiotracer administration in contrast to 24 hours in case of labeled leukocyte scan.

This is particularly advantageous in assessing the extent of retroperitoneal infection and its potential communication with the graft.

However, within the first months following graft implantation the specificity of ^18^F-FDG PET is reduced due to increased ^18^F-FDG uptake at sites of postoperative inflammatory changes. Further studies are needed for standardization of acquisition times and imaging interpretation criteria to establish the role of PET/CT in the diagnosis of graft infection [34–36].

The magnetic resonance imaging is not a first-line diagnosing tool of AEF because of its limited availability in the emergency setting and relatively long acquisition time.

In addition, signal voids resulting from periaortic gas collections and pulsation artifacts can cause considerable difficulties in image interpretation.

Transcutaneous ultrasonography has a very limited role in the diagnosis of AEF.

However, in hemodynamically unstable patients with a suitable body habitus it may enable fast detection of retroperitoneal hematoma or perigraft fluid.

According to the comprehensive literature review by Bergqvist and Björck the diagnostic accuracy of endoscopy in evaluation of AEF is low (30% accuracy of gastroscopy) [1].

Nevertheless endoscopy has its well established role as a primary tool in clinical management of gastrointestinal bleeding and allows to evaluate other than AEF etiologies of gastrointestinal hemorrhage [37–39]. Visualization of the third and fourth portions of duodenum is essential but frequently technically difficult.

At the present day conventional angiography is not a primary diagnostic modality in evaluation of AEF, but in certain clinical settings transcatheter arterial interventions may be useful in controlling the gastrointestinal hemorrhage related to AEF [39].

Without treatment, AEF has invariably catastrophic consequences with a mortality of virtually 100%. Conventional surgical treatment options are associated with high morbidity and mortality [40, 41] and include graft removal with primary or secondary axillobifemoral bypass as well as graft removal and in situ reconstruction, depending predominantly on the infection status of the surgical site [1, 42, 43].

The endovascular repair has emerged as a relatively new method of rapid hemorrhage control and restoration of peripheral perfusion in hemodynamically unstable patients. It can be regarded as a temporizing procedure prior to laparotomy, or a part of a long term palliation in high-risk surgical patients in whom the remained communication between the gut and aorta and the presence of infected prosthetic material preclude definitive eradication of infection [40, 41, 43].

## 3. Conclusion

The presence of aortoenteric fistula should be evaluated in any patient after conventional aortic reconstruction or endovascular aortic repair with gastrointestinal blood loss or with signs of graft infection.

Computed tomography is the most valuable diagnostic tool, although the observed abnormal CT findings are most frequently unspecific and similar to those secondary to perigraft infection. This fact underscores the importance of a strong clinical suspicion in management of this potentially lethal complication.
